# A new hypothesis for Parkinson’s disease pathogenesis: GTPase-p38 MAPK signaling and autophagy as convergence points of etiology and genomics

**DOI:** 10.1186/s13024-018-0273-5

**Published:** 2018-08-02

**Authors:** Julia Obergasteiger, Giulia Frapporti, Peter P. Pramstaller, Andrew A. Hicks, Mattia Volta

**Affiliations:** 1 Institute for Biomedicine, Eurac Research – Affiliated Institute of the University of Lübeck, Via Galvani 31, 39100 Bolzano, Italy; 2 Department of Neurology, General Central Hospital, Via Böhler 5, 39100 Bolzano, Italy; 3 Department of Neurology, University of Lübeck, Ratzeburger Allee, 23538 Lübeck, Germany

**Keywords:** Parkinson´s disease, alpha-synuclein, LRRK2, genetics, GWAS, p38, autophagy, lysosome, neuropathology, MAPK

## Abstract

The combination of genetics and genomics in Parkinson´s disease has recently begun to unveil molecular mechanisms possibly underlying disease onset and progression. In particular, catabolic processes such as autophagy have been increasingly gaining relevance as post-mortem evidence and experimental models suggested a participation in neurodegeneration and alpha-synuclein Lewy body pathology. In addition, familial Parkinson´s disease linked to LRRK2 and alpha-synuclein provided stronger correlation between etiology and alterations in autophagy. More detailed cellular pathways are proposed and genetic risk factors that associate with idiopathic Parkinson´s disease provide further clues in dissecting contributions of single players. Nevertheless, the fine-tuning of these processes remains elusive, as the initial stages of the pathways are not yet clarified.

In this review, we collect literature evidence pointing to autophagy as the common, downstream target of Parkinsonian dysfunctions and augment current knowledge on the factors that direct the subsequent steps. Cell and molecular biology evidence indicate that p38 signaling underlies neurodegeneration and autoptic observations suggest a participation in neuropathology. Moreover, alpha-synuclein and LRRK2 also appear involved in the p38 pathway with additional roles in the regulation of GTPase signaling. Small GTPases are critical modulators of p38 activation and thus, their functional interaction with aSyn and LRRK2 could explain much of the detailed mechanics of autophagy in Parkinson´s disease.

We propose a novel hypothesis for a more comprehensive working model where autophagy is controlled by upstream pathways, such as GTPase-p38, that have been so far underexplored in this context. In addition, etiological factors (LRRK2, alpha-synuclein) and risk loci might also combine in this common mechanism, providing a powerful experimental setting to dissect the cause of both familial and idiopathic disease.

## Background

Parkinson’s disease (PD) is the most common neurodegenerative movement disorder [1] and is defined by parkinsonism, a motor syndrome including resting tremor, bradykinesia, rigidity and postural instability. Non-motor symptoms (dementia, depression, sleep disorders) also manifest and can arise even before motor diagnosis. The pathology is associated with a profound loss of dopaminergic neurons in the Substantia Nigra pars compacta (SNc) [2, 3] accompanied by filamentous protein inclusions termed Lewy bodies (LBs) in the surviving neurons. LBs are mainly composed of alpha-synuclein (aSyn) and are found in both idiopathic and autosomal dominant, familial PD patients. These inclusions are the hallmark pathology not only in PD, but also a group of neurological disorders called synucleinopathies. Their pathogenic relevance is, however, still under debate [4]. Therapies that are capable of slowing down the progression of this disabling neurodegenerative disorder are currently not available and the mainstay of symptomatic treatment remains the pharmacological replacement of dopamine (DA) using its precursor L-DOPA [5]. Until 1997, the etiology of PD was essentially considered the result of several environmental factors such as viral infections, lifestyle and environmental pollution [6]. With the discovery that mutations and multiplications in the *SNCA* gene (coding for aSyn) are linked to familial PD, the first evidence of a genetic contribution to lifetime risk of PD was uncovered [7, 8]. These genetic alterations can also interact with environmental factors to contribute to disease onset [9]. The “genetics revolution” led to a redefinition of PD as a heterogeneous disorder comprising genetic implications (albeit in a limited percentage of patients), and prompted efforts on the investigation of the roles played by the genes involved. In addition to sequencing strategies in families with a history of PD, genome-wide association studies (GWAS) nominate several risk loci [10, 11]. The combination of all these molecular genetic methods holds promises to discover mechanisms underlying disease onset and/or progression. PD is a multifactorial disorder, the vast majority of cases are classified as idiopathic (i.e. of unknown etiology) and familial PD accounts for only ~10% of all PD cases worldwide [12]. Nevertheless, the study of gene mutations that segregate in families with PD offers a valuable opportunity to better understand molecular mechanisms, improve clinical diagnosis and inform trial design and neuroscientific modelling. Most of the past and recent discoveries in PD research point out three major cellular processes altered in the disease state: vesicle trafficking and synaptic transmission, autophagy and mitochondrial quality control. PD-related mutations are present since the embryonal life, but their pathophysiological effects are not evident until later in life (ranging between ~30 to 60 years of age, including early- and late-onset PD) and only in those patients who will develop PD [13, 14]. We speculate that cellular processes mentioned above might be part of a putative compensatory system and would be compromised with age. This view underlines the importance of understanding where and when these processes become dysfunctional. Notably, age is the major overall risk factor for PD [3]. This understanding could help to intervene in the very early stages of the disease and develop a disease-modifying treatment.

### Main text

In this review, we will focus on mechanisms involving aSyn and Leucine-Rich Repeat Kinase 2 (LRRK2), their impact on autophagy and we propose a novel perspective in the upstream modulation through Ras/Mitogen-activated Protein Kinase (MAPK) signaling. aSyn takes on a preponderant role in PD as a cause of familial disease, a genetic risk factor for idiopathic PD and the hallmark neuropathology. Mutations in LRRK2 are amongst the most common causes of familial disorder [15], displaying a clinical presentation that is mostly indistinguishable from idiopathic PD, and are found in 2-4% of idiopathic patients [16]. Both proteins play key functions in molecular mechanisms that are fundamental for the maintenance of neuronal viability, such as protein handling/degradation. These common pathways are coordinated, not only by familial genes, but also candidate genes predicted from identification in GWAS risk loci, suggesting alterations in more than one factor could addictively contribute to development of the disorder. Nevertheless, the same observation could indicate that the relative pathway *per se* is critical for pathogenesis, and individual perturbations along that pathway could be causal for the disease. Extensive research efforts have been conducted in the study of autophagy and elimination of aSyn aggregates, but no clear molecular pathway has been elucidated yet. Basing on genetics, genomics and functional biology, we specifically propose a novel hypothesis aimed at investigating an underexplored GTPase-p38 MAPK signaling in the modulation of autophagy that might regulate these cellular processes.

#### The autophagy-lysosome pathway: molecular mechanisms and implication in neurodegenerative diseases

As mentioned above, LBs, together with loss of DA neurons, represent the main pathological hallmark of PD [7, 17]. Although they are mainly composed of aggregated aSyn, other proteins like Tau, ubiquitine or amyloid-β (Aβ) are sometimes found at the neuropathological examination, leading to classification of PD as a proteinopathy. In healthy brains, these proteins are mostly present in a monomeric form, while in PD brains a conformational change occurs triggering the formation of small oligomers, which would eventually evolve into higher order structures. The moment when they become cytotoxic and the exact pathologic conformational shape are still controversial, but a growing body of evidence indicates insoluble aSyn as the pathogenic/pathologic molecular species [18, 19].

Generally, the steady-state levels of cellular proteins is controlled by their rates of production and degradation, but maintaining protein homeostasis in specialized secretory cells appears more challenging since the necessary and continuous synthesis of new proteins can cause cellular stress, eventually leading to proteotoxicity [20]. Therefore, either reducing production or stimulating degradation appeared to be promising strategies to prevent or revert uncontrolled aSyn accumulation [21]. An integrative view of quality control mechanisms in PD is emerging and centering the autophagy-lysosome pathway (ALP) as the process mostly involved in the clearance of pathologic aSyn [22, 23].

Autophagy is a powerful evolutionarily conserved catabolic process that mediates the degradation of unnecessary and dysfunctional proteins or organelles. Three types of autophagy have been identified: macroautophagy, microautophagy and chaperone-mediated autophagy (CMA). Macroautophagy remains the best characterized and will be referred to as autophagy hereafter. Microautophagy follows the same pathway as macroautophagy except from the direct sequestration of the cytoplasmic content by the lysosome. Finally, CMA is a highly selective process in which proteins with a specific amino acid motif are recognized and transported directly to the lysosome.

The process of autophagy begins with the formation of a double-membrane vesicle, termed autophagosome, through the extension of an isolation membrane (phagophore). This process can be broadly divided in two major steps regulated by distinct sets of autophagy-related genes (Atg): nucleation and elongation of the isolation membrane. The ULK (Unc-51 Like Kinase)/Atg1 kinase complex and the autophagy-specific phosphatidylinositide 3-kinase complex are important for the nucleation step, while Atg12- and Atg8/LC3-conjugation systems are essential for the elongation step [24]. Once Atg8/LC3 is lipidated and incorporated into the phagophore membrane, the growing membrane engulfs cytoplasmic content destined for degradation and closes in on itself. Then, the autophagosome may fuse with endosomes or directly combine with lysosomes to form autolysosomes, where hydrolases will dismantle the cargo into amino acids and other small molecules.

In neurons, autophagy appears highly compartmentalized. Within the soma, there is a population of autophagic vesicles derived from axons and synapses and a second population of vesicles generated in the cell body [25]. While the latter are created directly where their action will take place, the former are created at the distal tip of axons and are then transported by dynein towards the cell body following a retrograde transport [26]. The regulation of autophagy in cell models is well studied [27], but how the mechanisms work at the synapse, even more than a meter away from the cell soma, is still under investigation. Soukup and colleagues recently showed that the necessary proteins and mechanisms for autophagy are present at the synapse. EndophilinA (EndoA) is a presynaptic protein involved in vesicle endocytosis and colocalizes with autophagosomal markers. Phosphorylation of EndoA induces changes in the protein structure and in vitro this leads to formation of highly curved membrane zones, which are required at the elongation step of autophagosome formation [28].

In recent years, autophagy has been accepted as a cellular process deeply involved in the pathogenesis of both familial and idiopathic PD. Consistently, deletion of autophagic genes in mice causes locomotor defects, accumulation of polyubiquitinated proteins and neurodegeneration [29]. In addition, post-mortem evidence shows perturbation of autophagy markers in different neurodegenerative disorders [30–32]. Moreover, an important role for autophagy in brain is highlighted by the observation that this organ is the most severely affected in lysosomal storage disorders [33]. The most significant evidence of the negative impact of lysosome dysfunction emerged from the finding that heterozygous mutations in the *GBA* gene, encoding the lysosomal hydrolase Glucocerebrosidase (GCase), compromise its function of glucosylceramide cleavage and augment the risk of developing PD by approximately 20-fold [34]. Moreover, deletion of neuronal GCase in *Drosophila* causes dramatic lysosomal-autophagic defects [35]. Autophagosome number is increased and the level of the fly LC3 homologue is augmented, suggesting a block of the autophagic flux, consistent with observations in the SN of PD brains and associated to aSyn pathology [36]. Additionally, the autophagic machinery becomes less efficient with ageing in the healthy brain and stimulation of autophagy has been proposed to promote longevity [37, 38]. Induction of autophagy is an accepted experimental approach in PD and in neurodegenerative proteinopathies [39, 40], but might not always be sufficient. Neuropathological inclusions are capable of escaping degradation and impair the correct progression of the autophagic pathway [41]. A recent report also shows that accumulation of autophagosomes during lysosomal blockade leads to cytotoxicity [42], but this could also be a consequence of the impaired process as a whole.

Under physiological conditions, autophagy occurs at low levels, while this process is triggered under cellular stress, amino acid deprivation and presence of protein aggregates [24, 43], leading to both compromised autolysosomal activity and increased autophagosome synthesis [44]. However, defects in autophagosome-lysosome fusion alone are not sufficient to induce cellular toxicity, but in combination with increased autophagosome synthesis, they cause deleterious effects on cell viability. Consistently, increased number of autophagosomes has been observed in cultured cells challenged with parkinsonian neurotoxins, such as MPP^+^ (1-methyl-4-phenylpyridinium), rotenone and 6-OHDA (6-hydroxydopamine) [45].

Tackling the mechanisms that are altered and lead to a “pathway to disease” is the only option to progress to a real cure. Despite great advances in the understanding of autophagy, specific knowledge of the events taking place in a specific condition (such as PD) is critical and still lacking. In the following section, we discuss the role played by p38 MAPK in autophagy and neurodegenerative diseases, as this player has mostly been considered a general regulator of apoptosis but could hold novel insights for PD pathogenesis.

#### Involvement of p38 MAPK in autophagy and neurodegeneration

**Fig. 1:**
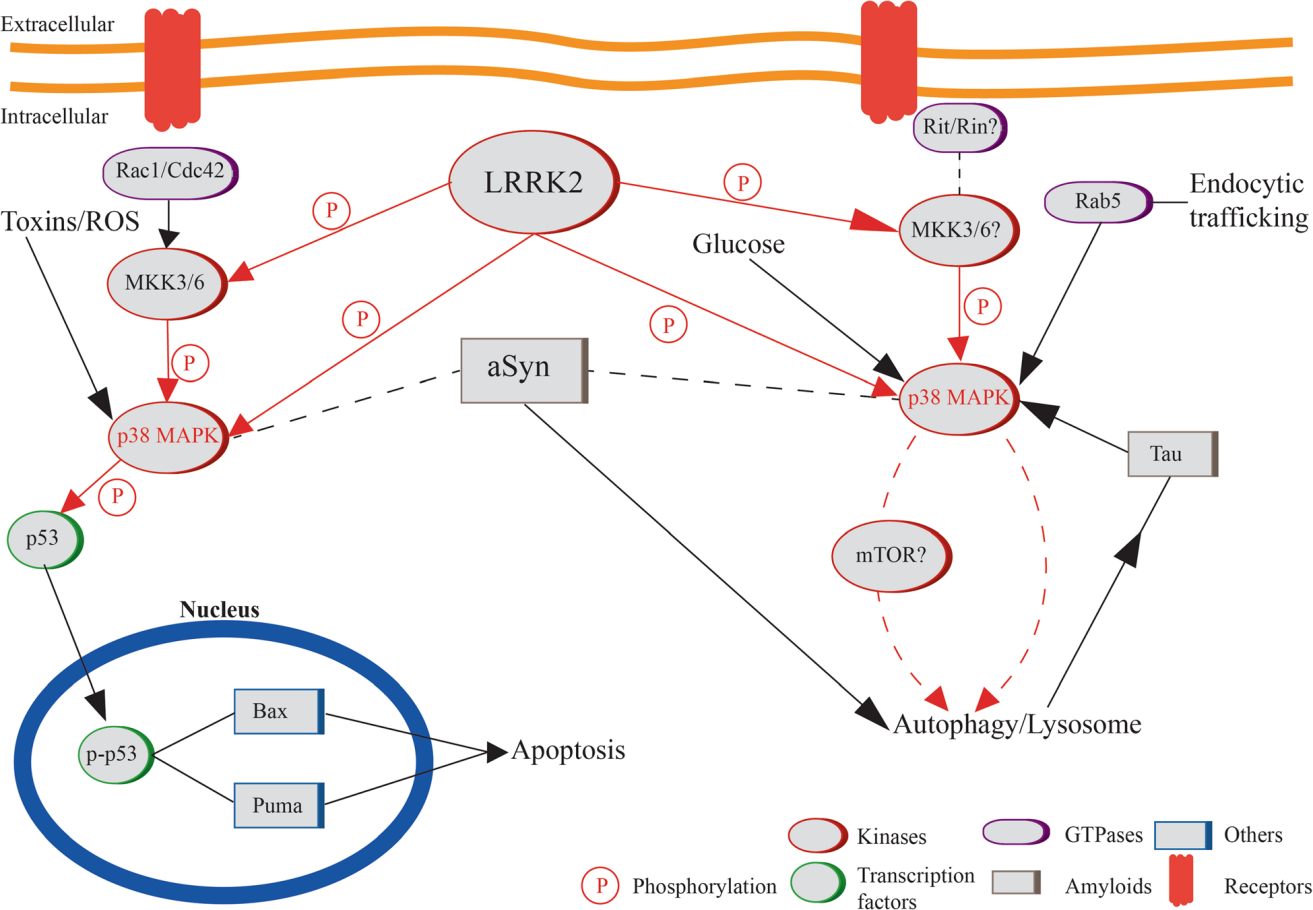
Molecular interactions between LRRK2, aSyn and p38 MAPK in the regulation of apoptosis and autophagy. Apoptosis and autophagy are the main cellular processes modulated by p38 MAPK. Upstream regulation appears similar, with membrane receptor stimulation coupling to Rho/Ras signaling, activation of MKKs and of p38. In the apoptotic pathway, p38 mainly targets p53 that, when phosphorylated, translocates to the nucleus and promotes gene transcription of the apoptosis mediators Bax and Puma. On the other hand, p38 could also modulate ALP with consequences on protein aggregate degradation (aSyn, Tau). LRRK2 and aSyn probably intervene in this pathway redirecting to one side or the other, depending on the stimuli. LRRK2 can phosphorylate MKKs and/or p38 directly, while aSyn might affect p38 activation through different mechanisms. The initial stages of the cascade, i.e. the specific GTPase (sub)family, could participate as director of the cellular pathway to be activated down the line

The p38 enzyme belongs to the MAPK family, together with ERK1/2 (Extracellular signal-Regulated Kinase), ERK5 and JNK (c-Jun N-terminal Kinase). They all signal through three modules: MAPK kinase kinases (MAPKKKs), MAPK kinases (MAPKKs) and MAPKs and are activated through dual phosphorylation of a tyrosine and a threonine residue (p-p38, pERK, pJNK) [46]. In mammals, four isoforms of p38 exist: α, β, γ, δ. They are divided into two groups, on one hand p38α and p38β, on the other p38γ and p38δ, mainly based on sequence homology and susceptibilities to different inhibitor compounds [47]. p38 MAPKs are primarily activated by environmental stresses and cytokines, but also growth factors. Two important kinases phosphorylating and thus activating p38 are Mitogen-activated Protein Kinase Kinase 3 and 6 (MKK3 and MKK6) [48] which are in turn activated by different MAPKKKs depending on the stimuli (Fig. 1). Upstream of MAPKKKs are small GTPases from the Rho subfamily (Rac1, Cdc242, Rho) and the Ras-like Rit subfamily [49–51].

The p38 MAPK signaling is known to be implicated in different neurodegenerative diseases through its regulatory action on apoptosis [52, 53]. In vivo, a p38 inhibitor prevents phosphorylation and translocation of p53 to the nucleus where it mediates transcription of Bax and Puma genes, which are known regulators of apoptosis. In vitro, p38 inhibition also prevents nuclear translocation of p53, similarly resulting in an enhancement of cell survival. [54].

In addition, recent research is shedding more light on the role of p38 MAPK in cellular processes other than apoptosis and suggests a role in autophagy. Available in vitro data do not consistently indicate the same direction of effect, which appears greatly dependent on the model system that is used (Table 1). Tang and colleagues show that p38 induces autophagy in astrocytes following accumulation of glial fibrillary acidic protein (GFAP), which is a principle filament protein in these cells. Mutations in the GFAP-coding gene cause Alexander disease, a neurodegenerative disorder affecting the brain white matter. Symptoms are mainly caused by defects in the myelin sheaths triggered by the accumulation of astrocytic GFAP [55, 56]. This accumulation is capable of activating p38, which in turn reduces mTOR phosphorylation and, as a consequence, leads to induction of autophagy [57]. This data suggests a cross-regulatory role on inflammation via autophagic modulation. A recent report indicates that p38 mediates microglial activation, but via a reduction of autophagy through phosphorylation-dependent inhibition of ULK1 [58]. Thus, an opposite effect on autophagy has been reported in microglia cells and astrocytes. In addition, it has been observed that hypo-osmotic conditions in hepatocytes activate p38, with a downstream suppression of the autophagic machinery [59]. Moreover, Webber et al show that depletion of p38α in HEK293 cells leads to a reduction in autophagy [60], whereas its activation in mouse embryonic fibroblasts produces an inhibition of the autophagosome-lysosome fusion [61]. These discrepancies might be caused by the cellular context in which studies are performed and thus, the direction of p38-mediated regulation of autophagy could be cell type-specific (Table 1).

**Table 1:** Participation of p38 MAPK signaling in ALP

Experimental context	Cell type	p38 MAPK signalling	ALP
Alexander disease	Astrocytes	↑	↑
Inflammatory process in nervous system	BV2 microglial cells	↑	↓
Hypo-osmotic condition	Hepatocytes	↑	↓
Depletion of p38 gene	HEK293 cells	↓	↓
Stimulation of p38 MAPK signaling	Mouse embryonic fibroblasts	↑	↓
Downregulation of p38 in AD	Neurons	↓	↑

The first two columns of the table indicate the model system and the cellular context in which studies are performed; in the last two columns the implication, seen as activation (↑) or suppression (↓), of p38 MAPK signaling and ALP in the disease/ experimental context, is shown

In early stages of Alzheimer´s disease (AD), defects in the ALP can be observed. Schnöder and colleagues were the first to show in vivo data on the possible impact of p38α on autophagy in AD, where Aβ peptide is produced through several digestive steps of amyloid precursor protein by the enzyme BACE1. The inhibition of BACE1 reduces Aβ load and therefore gained increasing attention for therapeutic drug development. Downregulation of p38α induces lysosomal degradation of BACE1 and a consequent reduction of Aβ generation. In addition, pharmacological inhibition of p38α increases autophagy [62, 63]. Thus, in AD activation of neuronal p38 seems to be detrimental for autophagy and underlie neuropathology. Activity of p38 MAPK is also under investigation in Huntington’s disease, where polyglutamine-expanded huntingtin activates p38 signaling in neurons and the overexpression of Mitogen-activated Protein Kinase/Dual-specificity Phosphatase 1 (MKP-1) prevents this activation, possibly having an impact on neuroprotection [64]. Of interest, immunohistochemical analysis of patient brain samples from PD and Dementia with Lewy Bodies reveals positive, granular and inclusion-like p-p38 staining, whereas control samples display only weak diffuse staining of p-p38. In addition, granular p-p38 staining is found in neurons of the SNc containing LBs or diffuse aSyn deposition [65]. Consistently, multiple evidence demonstrate that activation of p38 underlies the toxic effects of 6-OHDA and MPTP on DA neurons [54, 66–70]. These examples might indicate that p38 activity could underlie cellular damage when occurring in neurons. In this regard, it is important to bear in mind that p38 probably acts as an “effector”. We speculate that downstream consequences could also depend on the initial neurotoxic/neuropathological trigger (which would constitute the basis of an etiologic mechanism).

Based on these observations, we hypothesize p38 could participate in neurodegenerative disease pathogenesis also through modulation of autophagy. Nevertheless, how it could be involved specifically in PD etiology remains unclear and unexplored. We mentioned familial PD (especially autosomal dominant) could serve as a starting point to understand idiopathic PD and thus, in the following sections, we will discuss the possible relation of aSyn and LRRK2 with autophagy and p38 signaling.

#### Alpha-synuclein, autophagy and MAPK modulation

*SNCA* mutations (A30P, E46K, H50Q, G51D and A53T) and gene multiplications (duplication and triplication) have been identified by linkage analysis to cause rare forms of familial PD, at a rate of about 1% in different populations [8, 71–74]. Patients carrying *SNCA* mutations mostly develop early-onset PD and have a good response to L-DOPA in the first stages of the disease [75]. Disease progression of *SNCA* duplication patients is more similar to idiopathic PD cases [76], whereas *SNCA* triplication carriers usually have earlier onset and a more severe disease progression and these phenotypes appear correlated with aSyn protein levels. Recent GWAS studies confirmed strong association of the *SNCA* locus, supporting a role in the idiopathic disease as well [10, 11], with several polymorphisms across the whole locus that exhibit association to increased risk of PD [77].

The aSyn protein is ubiquitously expressed in the brain [78], but its precise function is largely unclear. It has been proposed to interact with the Soluble N-ethylmaleimide-sensitive factor (NSF) Attachment Protein Receptor (SNARE) complex and associate with synaptic vesicles in axon terminals, regulate their trafficking and neurotransmitter release [79]. Furthermore, aSyn participates in cargo sorting and protein degradation as it is involved in endosome trafficking, autophagy and CMA [8, 80–82]. In line with this, aSyn overexpression is known to have an impact on the autophagic machinery with an increase of autophagosomes, which is not paralleled by a matched increase in degradative capacity but rather an impairment [41, 83]. Moreover, literature evidence show that pathological aSyn reduces autophagy and causes lysosomal depletion in PD brains, cell and animal models [22, 36]. Physiologically, aSyn is degraded by both the ubiquitin proteasome system (UPS) and ALP [84, 85], but autophagy-mediated degradation appears to be reserved for higher molecular weight species, such as aSyn aggregates, that are not able to enter the proteasome [23]. This evidence tightly link lysosome biology and aSyn inclusions in PD pathophysiology, with the lysosome as the specific site where pathologic aSyn is delivered for degradation [86]. Hence, any dysfunction at this organelle might cause an accumulation of aSyn in the nervous system, which reflects what has been observed in knockout mice lacking cathepsin D, one of the major lysosomal proteases. PD caused by *GBA* mutations (see previously) also supports this view, as dysfunctional GCase enhances aSyn neuropathology [87].

In neurodegenerative diseases like PD, monomeric aSyn assembles into soluble oligomers or protofibrils, a process shown to be enhanced by the A30P and A53T mutations [88]. The formation of these protofibrils ultimately leads to the generation of insoluble amyloid fibrils. In addition it has been shown that aSyn oligomers are prone to be secreted via exosomes, a possible mechanism responsible for the abovementioned spreading of LB pathology [89] as cells and neurons can secrete aSyn via exocytosis [90, 91]. The exact underlying mechanisms are still under deep investigation, but this cell-to-cell transfer has been proposed to explain the spreading of LB pathology in experimental models and support the prion-like hypothesis of PD [92–94].

Overexpression of aSyn is cytotoxic in cell lines, but not necessarily causes the formation of inclusions [95]. At the same time, an effect on activation of MAPKs, such as p38, has been reported as overexpression leads to a reduction of their phosphorylation. At the same time aSyn overexpressing cells are more vulnerable to oxidative stress, which triggers the activation of p38 signaling. This is blocked by aSyn itself and thus leading to reduced cell viability [96]. Of note, Polo-like kinase 2 (Plk2) phosphorylates the Ser129 residue of aSyn in vitro, in primary neurons and in vivo. This result was confirmed by the reduction of pSer129-aSyn levels following knock-down of Plk2 [97]. When expressed at high levels, aSyn inhibits Plk2 function, which in turn leads to lower phosphorylation of the downstream substrates of Plk2, such as Rho guanine nucleotide exchange factors (GEFs) and GTPase activating proteins (GAPs), but also aSyn itself. In addition, aSyn inhibits p38 phosphorylation [96] and the dysregulated interaction between Plk2 and Rho GEFs/GAPs could result in reduced phosphorylation of p38 [98].

The amount of DA, produced by SNc neurons, is dramatically decreasing at a certain stage of the disease and accounts for motor symptoms. On the other hand, DA can exert toxic effects and is thought to contribute to neuronal loss [99, 100]. In a cell-based study, DA has been shown to induce a decrease in cell viability and an activation of p38 MAPK. In addition, aSyn expression was increased in this experimental setting and the autophagic machinery activated, which could be reversed by a p38 inhibitor. However, contrasting results have also been reported. Recently, neuronal overexpression of aSyn has been proposed not to activate p38, JNK or ERK1/2 [101], but replication of such data in a more quantitative way is needed.

In recent years, research focused on inflammatory processes that might be involved in PD pathogenesis. The contribution of glial cells and their release of inflammatory mediators is under current investigation. Interestingly, rapamycin- and trehalose-induced autophagy modulates phosphorylation of p38, which is activated by aSyn and required for inflammation-linked processes (which contribute to neurodegeneration [102]). Notably, both rapamycin and trehalose (autophagy enhancers) could reduce aSyn-dependent phosphorylation of p38 [103]. This collection of recent evidence suggests a novel link may be drawn between aSyn and p38 modulating cell physiology and survival via autophagy and not exclusively apoptosis [104] (Fig. 1). Additional experimental evidence is required to pinpoint the machinery of these interactions and the direction(s) of modulation.

#### LRRK2 in autophagy and MAPK signaling

LRRK2 is a large, multifunctional protein with a central catalytic GTPase/kinase core flanked by several protein-binding domains. Seven missense mutations, clustered in the Ras-of-complex (ROC) GTPase domain, C-terminal-of-ROC (COR) and kinase domains, segregate with PD in families [105]. These mutations show mild differences in their clinical presentation, but altogether, LRRK2 PD is basically indistinguishable from the idiopathic disorder, proposing familial LRRK2 PD as a window on idiopathic PD pathogenesis [15]. The G2019S substitution within the highly conserved kinase domain is associated to 4% of the autosomal dominant forms of familial PD and 1% of sporadic disease worldwide. At the adjacent residue, within the activation loop, a second mutation (I2020T) was discovered [106]. Both mutations are associated with a toxic gain of function, resulting in an increased kinase activity [107]. Three other confirmed pathogenic mutations have been described in the ROC domain affecting the same Arginine residue (R1441C/G/H), defining this codon as a hotspot for PD [108]. The majority of studies have reported these mutations to decrease GTP hydrolysis, increasing the residence time of the GTP-bound (active) state and indirectly enhancing kinase activity [109]. Nonetheless, the exact mechanism of the functional relationship between kinase and GTPase functions is still unresolved and one of the main controversial issues about LRRK2 biology [110].

Several substrates have been proposed to be directly phosphorylated by LRRK2, including LRRK2 itself, which complicated definition of a single cellular role [110]. Likely, LRRK2 acts in several physiological processes, of which vesicular trafficking and protein degradation have attracted specific research interest [111, 112]. Recent work revealed that members of the Ras analog in brain (Rab) family of small GTPases, involved in all forms of intracellular vesicular trafficking and sorting events, are key physiological phosphorylation substrates for LRRK2 [113, 114]. This is consistent with previous reports highlighting a role for LRRK2 in vesicle trafficking [115, 116], endolysosomal function and autophagy [117], and pointing out an important contribution to protein sorting and degradation.

Several studies show an involvement of LRRK2 in the regulation of autophagy and demonstrating that kinase activity modulates these effects, with consequences on PD pathogenesis and neuropathology [112, 118]. Of note, inhibition of LRRK2 kinase activity stimulates autophagy and regulates autophagosome formation [119]. At the presynaptic site, LRRK2 phosphorylates EndoA critically orchestrating the autophagic machinery in this neuronal compartment [40, 41]. Increased kinase activity due to the G2019S mutation also affects EndoA phosphorylation levels and, consequently, the regulation of synaptic autophagy [39]. Consistently, pathogenic LRRK2 mutations perturb lysosomal biology and morphology in a kinase-dependent manner. Overexpression of mutant LRRK2 in astrocytes causes an increase in lysosome size, number and function, a phenotype conserved in other cell types such as neuronal cell lines and human fibroblasts obtained from G2019S PD patients [120–123]. Conversely, Schapansky et al show a reduction in lysosomal size in G2019S KI primary neurons. These opposite results could be explained by the different models used, ranging from human non-neuronal cells to murine cultured neurons (mostly non-midbrain neurons) [124]. However, the average number of lysosomes per cell appears similarly changed, since it has been observed to increase in all cell models analyzed; a plausible explanation might be that upregulation of lysosome biogenesis serves as a compensatory mechanism. These data suggest that LRRK2 positively regulates the lysosomal system, but we cannot exclude that these changes might represent compensation to an insult in the pathway (e.g. autophagy inhibition). Nevertheless, the pathogenic G2019S mutation seems to exacerbate this function, but ultimately leading to lysosomal impairment [120].

As previously mentioned, the sometimes-contradictory literature and the complex nature of LRRK2 architecture complicate the definition of a role for LRRK2 and a conclusive demonstration of its cellular function(s). A physical and genetic interaction with Rab GTPases, helping to direct cytoplasmic LRRK2 to lipid membranes, has recently been uncovered, supporting the hypothesis of LRRK2 as a complexing scaffold [116]. Rab7L1, indicated by GWAS as a risk locus for idiopathic PD, functionally interacts with LRRK2 and regulates neurite outgrowth and trafficking of vesicles to the Golgi apparatus [122]. At the basis of this interaction, Beilina et al. further propose a complex able to function properly only when its components are present at the right stoichiometric ratio. These members are Rab7L1, Bcl-2 associated athanogene domain chaperone (BAG5) and cyclin G-associated kinase (GAK, notably another GWAS hit) and, when correctly combined with LRRK2, they promote autophagy-mediated clearance of Golgi-derived vesicles. In healthy individuals, a perfect orchestration of these factors is required and the impairment of a single component can affect the entire machinery. Mutations of LRRK2 gene do not affect the binding with its protein partners, but the overall function of the complex is compromised. Similarly, the formation of a partial complex is not sufficient for its correct function, suggesting a specific role for each protein within the complex [125]. This evidence strongly support the hypothesis that physical interactions between different PD-causing genes (e.g. LRRK2) and/or PD-associated factors (e.g. Rab7L1 and GAK) influence intracellular trafficking and autophagy to modulate disease expression (onset and progression). Additionally, other classical Rho GTPases (such as Cdc42, RhoA, Rac1) were found to bind LRRK2, with the strongest specificity of interaction shown by selective co-immunoprecipitation of LRRK2 with Rac1 [126]. Co-expression of LRRK2 and Rac1 increases the activity of the latter, directing it to its site of action. Rac1 is important in modulating actin cytoskeletal dynamics, which is required for the maintenance of neurite morphology.

Thus, similar to aSyn, LRRK2 appears to be involved in GTPase signaling to regulate downstream cellular processes. This could be a direct process (i.e. PD-related mutations directly alter GTPase regulation), but the possibility exists that other pathways and/or upstream regulators are present. Effects of LRRK2 also appear dependent on the cell type under investigation and likely additional, more indirect mechanisms might be involved. Several studies have identified pathway candidates to be activated by LRRK2, which is also reported to play a role in MAPK signaling cascades, not acting as the classical modulator of MAPK phosphorylation activity, but rather inducing a series of events leading to changes in cell physiology (Fig. 1). Binding of LRRK2 to MKK-3/6 and MKK-4/7 was found to mildly activate p38 and JNK pathways [127–129]. LRRK2 variants associated with a gain in kinase activity, such as G2019S, display a 3-4 fold increase of MKK phosphorylation compared to wild-type (WT) LRRK2. In addition, treatment with a selective ERK inhibitor suppresses the upregulated transcription of aSyn observed in WT LRRK2 overexpressing cell lines [130]. Consistently, application of a MEK inhibitor blocks the pathologic effect on autophagy and neurite shortening reported in several LRRK2 mutations [131]. Importantly, LRRK2 mediates the cytotoxic increase in p-p38 and the G2019S mutation causes sustained p38 activation and neurodegeneration in vivo [132].

We hypothesize that MAPKs provide a group of potential targets and/or interactors that could link neuronal toxicity, cytoskeletal dynamics and vesicular transport of mutant LRRK2 to molecular mechanism of autophagy. This pathway could well overlap or act in parallel to other, more established mechanisms such as regulation of Rab GTPases. In the following section, we discuss further details on common mechanisms and how they could impinge on protein degradation and underlie PD onset.

#### Interactions of aSyn and LRRK2 with autophagy and convergence on GTPase-p38 MAPK: a new working hypothesis for PD?

These numerous examples suggest that ALP could constitute a “*unifying theme*” in PD [86]. Nevertheless, the upstream regulation of ALP and the level at which the dysfunction is triggered is far from being elucidated. Novel mechanisms are being explored, combining different PD-related proteins into a common pathway. For example, LRRK2 mutations enhance protein levels of the lysosomal cation pump ATP13A2 [120]. Loss of function mutations in its coding gene are associated with parkinsonian Kufor-Rakeb syndrome, trigger alkalinisation of lysosomes and diminished efficiency of hydrolysis [133]. The G2019S mutation induces lysosomal deficits in different cell models [134] and upregulation of ATP13A2 could represent a compensatory mechanism as overexpression of ATP13A2 is thought to rescue lysosome dysfunction [135]. This evidence suggest a minimum cell-autonomous mechanism where LRRK2 pathogenic mutations alter lysosome function and aSyn homeostasis in cultured neurons [124].

More detailed interactions begun to unveil, as the role of LRRK2 in aSyn pathology gained strong interest following the observation that neurodegeneration elicited by virally delivered aSyn is alleviated by LRRK2 deletion [136] and kinase inhibition [137]. In addition, overexpression of the pathogenic LRRK2-G2019S enhances aSyn pathology triggered by synthetic aSyn pre-formed fibrils (PFFs) [138], which is reduced by acute LRRK2 silencing in vivo [139]. Interestingly, aSyn and LRRK2 show pro-inflammatory activity in microglial cells [140, 141], where p38-dependent inhibition of autophagy also mediates inflammatory actions (as previously discussed). Prolonged LRRK2 kinase inhibition in astrocytes leads to autophagy induction via hyperphosphorylation of ULK1 [142] on a distinct residue than the ones phosphorylated by p38 MAPK in microglia [58]. These observations add additional convergence points to the hypothesized pathway.

Collectively, this evidence show that aSyn pathology can be modulated by LRRK2 by an unknown mechanism. The common impingement on endosomal trafficking and autophagy, added to the prominent role of the latter in aggregate clearance, strongly points to regulation of protein degradation pathways as the cellular substrate where they probably meet. However, the fine modulation of such mechanisms is largely unexplored and the contribution of other players is still missing. Clarifying these still obscure details much likely will hold novel insights for pathogenesis and disease-modifying treatments. In the previous sections, we presented evidence that both LRRK2 and aSyn interact with MAPKs and p38 but, despite promising, these observations do not provide information on the mechanisms of activation and regulation of signaling. Other players must be involved and, following the directions suggested by genetics and genomics interactions detailed in the previous sections, we hypothesize genes/loci indicated in PD GWAS could shed light on these processes. We speculate that Ras/Rho-p38 MAPK signaling might play a decisive role in connecting dysfunctional proteins linked to familial PD and ALP in one pathway to disease. In addition, the contribution of genetic risk factors might explain pathogenesis in idiopathic disease as well. In this context, we dissected the implication of each GWAS hit in both MAPK pathway and ALP. In Table 2 we summarized the possible interaction of each most recently associated candidate gene [10, 11]. Most of these are of no clear cellular function and implication in p38/MAPK signaling has been scarcely investigated. Nevertheless, a few genes are directly linked to p38 signaling, supporting this as the connecting link between aSyn and LRRK2. With this hypothesis in mind, further upstream regulation could delineate the whole process and, at this level, GWAS could provide novel indications.

**Table 2:** Genes associated to PD, their involvement in p38/MAPK signaling and ALP

SNP	Candidate gene	Implication in p38 MAPK signaling	Implication in ALP
rs35749011	GBA	Yes (activation of p38 MAPK signaling in vivo and in vitro) [154]	Yes (deletion causes lysosomal-autophagic defects) [35]
	**SYT11**	Not studied	Yes (required for lysosome exocytosis) [155]
rs823118	NUCKS1	Not studied	Not studied
	SLC41A1	Yes (activated by p38 MAPK signaling) [156]	Not studied
	**RAB7L1**	Not studied	Yes (promotes clearance of Golgi-derived vesicles) [125]
rs10797576	SIPA1L2	Not studied	Not studied
rs6430538	TMEM163	Not studied	Not studied
	CCNT2	Not studied	Not studied
	**ACMSD**	Not studied	Not studied
rs1474055	STK39	Yes (activation of p38 MAPK signaling) [157]	Not studied
rs115185635	CHMP2B	Yes (modulation of p38 MAPK signaling) [158]	Yes (implicated in vital membrane deformation functions in autophagy) [159]
	**KRT8P25**	Not studied	Not studied
	**APOOP2**	Not studied	Not studied
rs12637471	MCCC1	Not studied	Not studied
rs34311866	TMEM175	Not studied	Yes (setting lysosomal membrane potential and maintaining pH stability) [160, 161]
	DGKQ	Yes (modulation of p38 MAPK signaling in different cell types) [162, 163]	Not studied
	**GAK**	Possible implication in p38 MAPK signaling [164]	Yes (implication in lysosomal enzyme sorting, clearance of Golgi-derived vesicles and modulation of autophagic flux) [125, 165]
rs11724635	FAM200B	Not studied	Not studied
	CD38	Yes (implication in p38 MAPK signaling in T cells) [166, 167]	Yes (role in autophagosome trafficking and lysosomal function) [168, 169]
	**BST1**	No phosphorylation of MAPKs in mouse macrophages [170]	Not studied
rs6812193g	FAM47E	Not studied	Not studied
	**SCARB2**	Yes (activation of p38 MAPK signaling) [171]	Yes (implication in biogenesis and reorganization of endosomes and lysosomes [172]
rs356182	SNCA	Yes (inhibition of p38 phosphorylation) [96, 98]	Yes (implication in autophagy) [22, 36, 41, 83]
rs9275326	HLA-DRB6	Not studied	Not studied
	HLA-DQA1	Not studied	Not studied
	**HLA-DQB1**	Not studied	Not studied
rs199347	KLHL7	Not studied	No (mediation of protein degradation via UPS) [173]
	NUPL2	Not studied	Not studied
	GPNMB	Possible involvement in p38 MAPK signaling [174–176]	Yes (involvement in LC3 recruitment to the phagosome) [177]
rs591323	MICU3	Not studied	Not studied
	**FGF20**	Yes (activation of MAPK signaling) [178, 179]	Not studied
rs117896735	BAG3	Yes (p38 MAPK signaling activates BAG3 transcription) [180, 181]	Yes (mediation of selective autophagy) [182–184]
	**INPP5F**	Not studied	Not studied
rs3793947	DLG2	No (target of ERK2) [185, 186]	Not studied
rs329648	MIR4697	Not studied	Not studied
rs76904798h	LRRK2	Yes (activation of p38 MAPK signaling) [127–129]	Yes (implication in autophagy) [112]
rs11060180	OGFOD2	Not studied	Not studied
	**CCDC62**	Not studied	Not studied
rs11158026	GCH1	Yes (interaction with p38 MAPK signaling) [187, 188]	Not studied
rs1555399	TMEM229B	Not studied	Not studied
rs2414739	VPS13C	Not studied	Not studied
rs14235	ZNF646	Not studied	Not studied
	KAT8	Not studied	Yes (regulation of the outcome of autophagy) [189–191]
	**BCKDK**	No (upregulation of MEK/ERK MAPK signaling) [192]	Not studied
	**STX1B**	Not studied	Not studied
rs17649553	ARHGAP27	Not studied	Not studied
	CRHR1	Yes (activation of ERK and p38 MAPK signaling) [193, 194]	Not studied
	SPPL2C	Not studied	Not studied
	MAPT	Yes (hyperphosphorylation mediated by p38 MAPK signaling) [195–198]	Yes (implication in autophagic pathway) [199, 200]
	STH	Not studied	Not studied
	KANSL1	Not studied	Not studied
rs12456492	SYT4	Not studied	Not studied
	**RIT2**	Yes (implication in p38 MAPK signaling) [149, 150, 153, 201, 202]	Not studied
rs62120679	LSM7	Not studied	Not studied
	**SPPL2B**	Not studied	Not studied
rs8118008	DDRGK1	Not studied	Possible implication in ALP [203]
rs4653767	ITPKB	Not studied	Not studied
rs34043159	IL1R2	Yes (p38 inhibition prevents IL1r2 mRNA expression) [204]	Not studied
rs353116	SCN3A	Yes (possible co-regulation of p38 MAPK pathway and SCN3A) [205, 206]	Not studied
rs4073221	SATB1	Yes (possible co-regulation of p38 MAPK pathway and SATB1 protein) [207]	Not studied
rs12497850	NCKIPSD	No (interaction with ERK1) [208]	Yes (possible implication in ALP) [209]
	CDC71	Not studied	Not studied
rs143918452	ALAS1	Not studied	Not studied
	TLR9	Yes (activation of p38 MAPK signaling)[195, 210, 211]	Yes (stimulation of autophagy) [212–214]
	DNAH1	Not studied	Not studied
	BAP1	Not studied	Not studied
	PHF7	Not studied	Not studied
	NISCH	Yes (activation of p38 MAPK signaling) [215]	Yes (upregulated when autophagy is perturbed) [216]
	STAB1	Not studied	Yes (localization to late endosomes and lysosomes) [217]
	ITIH3	Not studied	Not studied
	ITIH4	Not studied	Not studied
rs78738012	ANK2	Yes (activation of p38 MAPK signaling) [218]	Yes (implication in lysosome transport) [219]
	CAMK2D	Not studied	Not studied
rs2694528	ELOVL7	Not studied	Not studied
rs9468199	ZNF184	Not studied	Not studied
rs2740594	CTSB	Not studied	Yes (lysosomal protease) [220–222]
rs2280104	SORBS3	Not studied	Not studied
	PDLIM2	Not studied	Not studied
	C8orf58	Not studied	Not studied
	BIN3	Not studied	Not studied
rs13294100	SH3GL2	Not studied	Not studied
rs10906923	FAM171A1	Not studied	Not studied
rs8005172	GALC	Not studied	Yes (lysosomal enzyme) [223–225]
rs11343	COQ7	Not studied	Yes (inhibition of autophagy) [226]
rs4784227	TOX3	Not studied	Not studied
rs601999	ATP6V0A1	Not studied	Yes (V-ATPase subunit) [227–230]
	PSMC3IP	Not studied	Not studied
	TUBG2	Not studied	Not studied

The first column of the table shows loci identified in GWAS conducted by Nalls et al (2014), incorporated with 17 new loci emerged by the last GWAS conducted by Chang et al. (2017). Loci reported only by Nalls et al are represented in bold. Each locus is coupled with a candidate gene associated with the development of PD. In the third column, the role of each gene in MAPK signaling, in particular in p38-MAPK signaling, is shown. In the fourth column, involvement of the candidate gene in ALP is reported

Involvement of small GTPases in PD pathogenesis is a current focus of research that has indicated their participation in several cellular processes that are altered in PD (for a recent review see [143]). With regards to our focus, LRRK2 phosphorylates two specific Rab GTPases [113]. Rabs are essential regulators of the endosome system, which mediates intracellular trafficking and autophagy [144]. Their role in endoplasmic reticulum-Golgi trafficking has also been investigated in PD with reports of alterations caused by aSyn [145, 146]. Previously, we mentioned that the Rac1 member of the Rho family of GTPases interacts with LRRK2. Recently, activity of Rac1 has been reported to be critical in the maintenance of DA neurons and to counteract pathologic aSyn via modulation of autophagy [147]. We also described how aSyn dysregulates Plk2-dependent modulation of Rho GEFs and GAPs. This could also impact aggregation of aSyn as Plk2 has been nominated as the kinase responsible for the Ser129 phosphorylation of aSyn in the same study [98]. These examples illustrate the multifaceted connection of GTPases to PD pathogenic processes.

We have recently observed that LRRK2 and aSyn dysregulate expression of the *RIT2* gene (*Obergasteiger et al., in preparation*), which could underlie novel cellular mechanisms to be explored. Genomic variability in its locus has been recently reported [10, 148] to increase risk for PD. The *RIT2* gene codes for the protein Rin that, together with Rit, is a member of the Rit subfamily of Ras-like small GTPases. Their cellular functions have been poorly investigated, but an emerging interest for the processes under examination might emerge. Indeed, Rin is involved in neuronal differentiation, p38 and ERK signaling pathways downstream of nerve growth factor (NGF) signaling, but its role in PD is still unknown [149]. Rin is expressed selectively in neural tissue [150], is enriched in rodent DA neurons [151] and shows reduced expression in the remaining SNc of PD brains [152]. Both Rit-like GTPases (Rit and Rin) were shown to regulate p38 MAPK signaling in cells in response to oxidative stress [153] and Rin specifically activates neurotrophin-mediated p38α MAPK signaling [150]. Preliminary results on Rin function and its connection to p38 are available, but definitive interpretation is still prevented by the paucity of the published data. In particular, further studies would need to causally join Rin-p38 activation with LRRK2 and/or aSyn function in the modulation of protein degradation. Moreover, more suitable cellular models, like primary or iPSC-derived neurons, are required to bear a stronger impact in the context of PD.

## Conclusions

From our literature analysis, we hypothesize that p38 MAPK signaling might be a key process involved in PD and could be at least one of the “missing links” between most known cellular players and autophagy (Fig. 1). However, its specific role in disease pathogenesis needs further clarification and targeted experimental validation to dissect its contribution from the other MAPKs (if any). We propose p38 MAPK signaling may regulate autophagy, which has gained a priority role in PD research [11]. We also speculate that the Rin protein, upstream of p38 MAPK, could contribute to the orchestration of the autophagic machinery. The further investigation of autophagy- and/or MAPK-related GWAS genes could provide additional information on novel cellular processes contributing to PD onset and progression, which can eventually be targeted for PD therapy.

## Abbreviations

6-OHDA: 6-hydroxydopamine
AD: Alzheimer´s disease
ALP: Autophagy lysosome pathway
aSyn: Alpha-synuclein
Atg’s: Autophagy-related genes
Aβ: Amyloid-β
BAG5: Bcl-2 associated athanogene domain chaperone
CMA: Chaperone-mediated autophagy
COR: C-terminal-of-ROC
DA: Dopamine
EndoA: EndophilinA
ERK: Extracellular Signal-regulated Kinase
GAK: Cyclin-G-associated kinase
GAPs: GTPase activating proteins
GCase: Glucocerebrosidase
GEFs: Guanine nucleotide exchange factors
GFAP: Glial fibrillary acidic protein
GWAS: Genome-wide association studies
JNK: c-Jun N-terminal kinase
LBs: Lewy bodies
LRRK2: Leucine-Rich Repeat Kinase 2
MAPK: Mitogen-activated protein kinase
MAPKKKs: MAPK kinase kinases
MAPKKs: MAPK kinases
MKK-3/6: Mitogen-activated protein kinase kinases 3/6
MKK-4/7: Mitogen-activated protein kinase kinases 4/7
MKP-1: Mitogen-activated protein kinase/dual-specificity phosphatase 1
MPP^+^: 1-methyl-4-phenylpyridinium
NGF: Nerve growth factor
NSF: N-ethylmaleimide-sensitive factor
PD: Parkinson’s disease
PFFs: Pre-formed fibrils
Plk2: Polo-like kinase 2
Rab: Ras analog in brain
ROC: Ras-of-complex
SNARE: Soluble NSF Attachment Protein Receptor
SNc: Substantia Nigra pars compacta
ULK: Unc-51 Like Kinase
UPS: Ubiquitine proteasome system
WT: Wild-type
